# Amiodarone and Ravuconazole Combination Potentiates Chemotherapy against Drug-Resistant Trypanosoma cruzi Strains

**DOI:** 10.1021/acsomega.5c00996

**Published:** 2025-05-16

**Authors:** Breno Vilas Boas Raimundo, Ana Lia Mazzeti, Maria Terezinha Bahia, Ivo Santana Caldas, Valquíria Ângelis Fernandes, Elda Gonçalves Santos, Rodrigo Dian de Oliveira Aguiar Soares, Aline Silva de Miranda, Rômulo Dias Novaes, Lívia de Figueiredo Diniz

**Affiliations:** † Laboratório de Parasitologia Básica, Departamento de Patologia e Parasitologia, Instituto de Ciências Biomédicas, 74347 Universidade Federal de Alfenas, Alfenas 37130-000, Minas Gerais, Brazil; ‡ Laboratório de Parasitologia Básica e Aplicada, Departamento de Ciências Biomédicas e Saúde, Universidade do Estado de Minas Gerais, Passos 37900-106, Minas Gerais, Brazil; § Núcleo de Pesquisas em Ciências Biológicas, Universidade Federal de Ouro Preto, Ouro Preto 35400-000, Minas Gerais, Brazil; ∥ Departamento de Biologia Estrutural, Instituto de Ciências Biomédicas, Universidade Federal de Alfenas, Alfenas 37130-000, Minas Gerais, Brazil; ⊥ Laboratório de Neurobiologia “Conceição Machado”, Departamento de Morfologia, Instituto de Ciências Biológicas, 28114 Universidade Federal de Minas Gerais, Belo Horizonte 31270-901, Brazil

## Abstract

Drug combinations represent valuable opportunities for developing more effective and safer treatments for Chagas disease. In this study, we investigated the effects of ravuconazole and amiodarone combinations against Trypanosoma cruzi infection. After ruling out antagonistic effects and increased cytotoxicity in vitro, a short-term in vivo protocol was used to evaluate parasitemia, immune response, and heart damage caused by the Y and Colombian T. cruzi strains in BALB/c and Swiss mice. Untreated mice displayed elevated parasitemia and pro-inflammatory cytokine levels, with strain-specific differences in IL-10 levels and the presence of parasites in the myocardium. Amiodarone monotherapy failed to exhibit in vivo anti-T. cruzi activity. While ravuconazole monotherapy at a subtherapeutic dose reduced parasitemia in all treated animals, its effects on the host response varied depending on the mouse and parasite strain, demonstrating greater activity in Swiss mice infected with the Y strain. Remarkably, amiodarone enhanced ravuconazole efficacy as combination chemotherapy was more effective than monotherapy in downregulating parasite load and pro-inflammatory cytokine levels, attenuating cardiac damage in all experimental models. These findings demonstrate a positive in vivo interaction between ravuconazole and amiodarone against T. cruzi infection. Furthermore, they highlight the relevance of the host genetic background and parasite strain on treatment outcomes.

## Graphical abstract

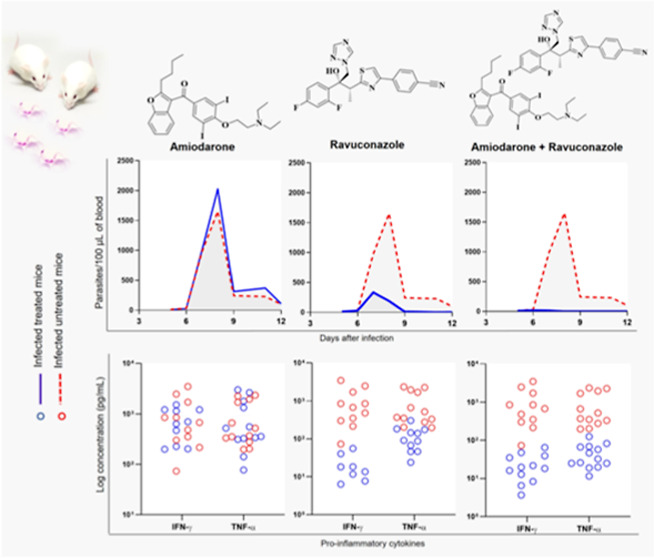

## Introduction

The first-line treatment for Chagas disease is based on benznidazole and nifurtimox, nitro heterocyclic drugs that frequently fail to cure patients in the chronic phase of disease, which is the most prevalent.^1^ Both drugs are administered as long therapeutic regimens of 8 weeks and induce considerable adverse events that determine treatment discontinuation and therapeutic failure.^2^ These limitations emphasize the urgent need to develop safer and more effective chemical entities, especially considering that Chagas disease remains determining significant morbidity and mortality rates in Latin American and nonendemic areas (e.g., Europe, North America, and Western Pacific).^3^

Despite the large number of potential preclinical drug candidates for Chagas disease over the past 3 decades, few proved in vivo efficacy against Trypanosoma cruzi infection.^4,5^ The inhibitors of T. cruzi CYP51 posaconazole and ravuconazole as well as the 2-nitroimidazole fexinidazole show promising experimental antiparasitic effects. However, they presented limited against human Chagas disease. Accordingly, CYP51 inhibitors failed to induce parasitological cure in the asymptomatic chronic phase,^4–7^ and the therapeutic doses of fexinidazole did not have acceptable safety, so its use as monotherapy was stopped.^8^

Considering the scarcity of new promising anti-T. cruzi drug candidates and the main drawbacks of nitroderivative-based treatments, strategies based on repositioning and drug combinations have been investigated to improve the efficacy and tolerability of anti-T. cruzi chemotherapy.^5^ The number of studies addressing these strategies has grown substantially in the last 15 years, including clinical trials.^5,6,9–15^ Although these efforts have not yet been translated into new therapeutic alternatives, they were critical in highlighting key issues related to preclinical models of Chagas disease and the challenges in advancing preclinical candidates to a clinical context.^16,17^ However, discussions about models for evaluating drug combinations remain notably scarce.^13^

In this work, we investigated the anti-T. cruzi potential and the impacts of host response resulting from the combined treatment using repositioned drugs, ravuconazole and amiodarone, on different experimental models. Amiodarone (Figure 1A), a benzofuran derivative, is a class III antiarrhythmic used clinically, including symptomatic Chagas disease treatment.^20^ Previous preclinical data had supported the activity of amiodarone against T. cruzi by disrupting intracellular Ca^2+^ and interference with ergosterol biosynthesis via oxidosqualene cyclase inhibition.^21,22^ Ravuconazole (Figure 1B) is a triazole antifungal that disrupts ergosterol biosynthesis by inhibiting C14α sterol demethylase, showing potent anti-T. cruzi activity in preclinical studies^18,23^ and in clinical trials.^8,12^ However, it has not achieved curative effects with the tested therapeutic regimens. To enhance azole effectiveness, investigating synergistic compounds has become a key research focus.^24^ Notably, some studies have shown that amiodarone can act synergistically with different azole derivatives against fungi^24^ and T. cruzi strains susceptible and partially resistant to benznidazole.^22,26^

**Figure 1:**
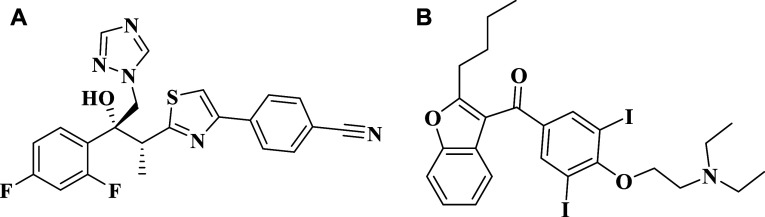
Chemical structures of ravuconazole (A) and amiodarone (B).

Recognizing a valuable opportunity to provide benchmarks for preclinical screening of drug combinations and develop more efficient antiparasitic therapeutic regimens, we used a comprehensive approach involving mouse lineages with different patterns of T. cruzi susceptibility and parasite strains with variable pharmacological resistance to investigate the pharmacological potential of ravuconazole and amiodarone combination to control parasite load, immune response, and tissue damage in experimental Chagas disease.

Our study aims to contribute to the development of rapid and reliable acute-phase models that can guide the prioritization of promising combinations for subsequent evaluation in chronic models, thereby accelerating the drug development pipeline.

## Results

### Ravuconazole and Amiodarone Show Additive Effect on T. cruzi Amastigotes In Vitro without Increased Cytotoxicity

First, we determined the toxicity profile of drug combinations on host cells and then the nature of the interaction between amiodarone and ravuconazole against the clinically relevant amastigotes forms of the Y T. cruzi strain. Our data indicate toxicity associated with amiodarone in a concentration-dependent manner (Figure 2A), with a CC-50 value of 28 μM in the H9c2 model. In contrast, ravuconazole did not induce toxic effects at any of the tested concentrations or exacerbate the toxicity of amiodarone (Figure 2A). When tested in combination in a 1:1 rate against intracellular amastigotes, amiodarone and ravuconazole showed an additive effect, with FIC values of 1.62 and 1.37 for EC_50_ and EC_90_ levels, respectively (Figure 2B,C).

**Figure 2:**
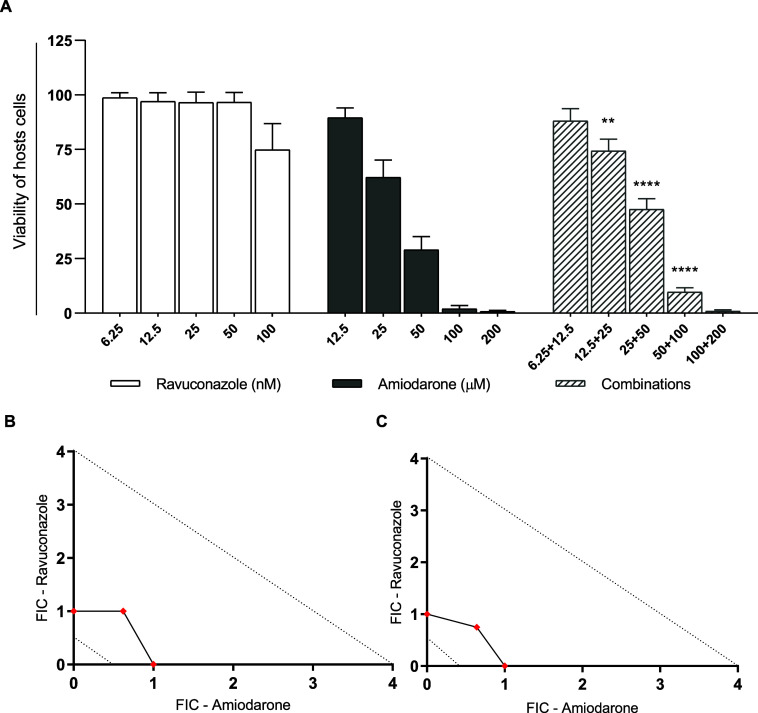
Effects of ravuconazole and amiodarone combination on host and parasites. (A) Viability of uninfected H9c2 cells incubated with ravuconazole and amiodarone, individually and in combination, for 48 h. **p < 0.01; ****p < 0.0001 compared to the same concentration of amiodarone in monotherapy. (B,C) Isobolograms representing the absence of interaction between ravuconazole and amiodarone against intracellular amastigotes of the Y T. cruzi strain at EC_50_ (B) and EC_90_ (C) levels. The dotted lines indicate the theoretical thresholds for synergism (<0.5) and antagonism (>4).

### Amiodarone Enhances the Effect of Ravuconazole on T. cruzi Infection in BALB/c and Swiss Mice Infected with Y Strain

To investigate whether amiodarone could potentiate the effect of ravuconazole in vivo and explore the impact of the experimental models on combination therapy outcomes, we developed a short-term evaluation optimized model, testing two different mouse lineages, BALB/c and Swiss, infected with the highly virulent Y T. cruzi strain. Figure 3 shows the parasitemia curves resulting from both mice lineages infected with the Y T. cruzi strain and the area under the parasitemia curve (AUC) as an objective indicator of parasite load.

**Figure 3:**
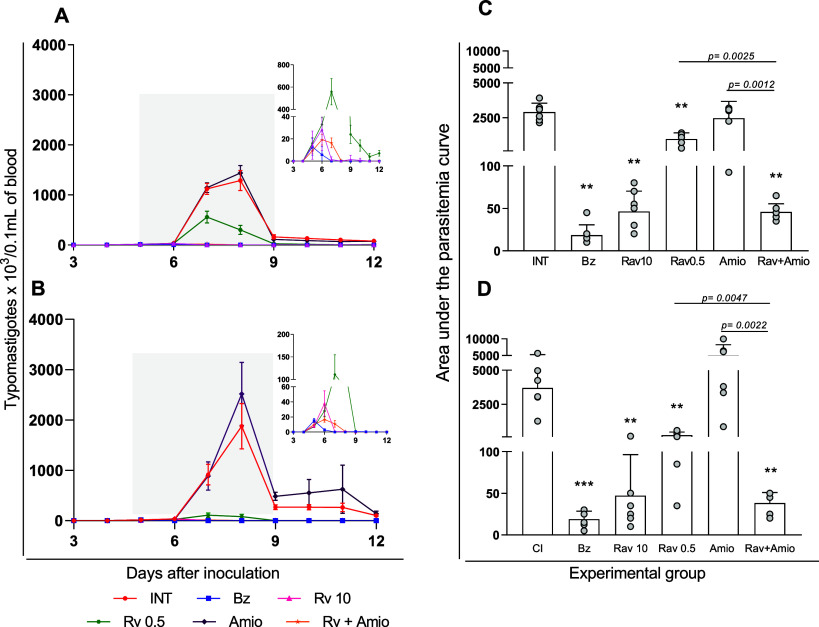
Effects of ravuconazole and amiodarone combination on a benznidazole partially resistant T. cruzi strain. Parasitemia curves and areas under parasitemia curves for BALB/c (A,C) and Swiss (B,D) mice infected with Y T. cruzi strain and treated with benznidazole at 100 mg/kg body weight (Bz); ravuconazole at 0.5 mg/kg (Rav 0.5); ravuconazole at 10 mg/kg (Rav 10); amiodarone at 50 mg/kg (Amio) or a combination of ravuconazole 0.5 mg/kg and amiodarone 50 mg/kg (Rav + Amio). Infected untreated controls (INT) received water. Treatments were administered orally from the 5th to the 9th day after infection. Insets in A and B show parasitemia curves excluding mice treated with amiodarone and untreated controls. Data are presented as mean ± SD. Asterisks indicate significant differences compared to infected untreated mice. *p < 0.05, **p < 0.01, ***p < 0.001. *p*-Values for comparisons between combination therapies and monotherapies at equivalent doses are shown in the graphs.

Parasitemia peaked at 7–8 days postinoculation, with mean maximum levels of 1385 and 1879 blood trypomastigotes/μL for BALB/c and Swiss mice, respectively (Figure 3A,B). Benznidazole at the reference dose reduced parasitemia to levels below the detection limit. Amiodarone at 50 mg/kg alone had no effect on parasitemia in either lineage. Ravuconazole showed a dose-dependent anti-T. cruzi effect: at the reference dose of 10 mg/kg, it suppressed parasite proliferation, as evidenced by decreased AUC (Figure 3C,D). At lower dose (0.5 mg/kg), ravuconazole’s trypanocidal effect varied by lineage, being significantly more potent in *Swiss* infected mice (reducing AUC by 16-fold compared to 2.5-fold in BALB/c mice), though it reduced parasitemia in both groups compared to untreated controls (Figure 3C,D). Notably, the combination of ravuconazole and amiodarone was more effective than either monotherapy at the same doses, reducing parasitemia levels in both host models (Figure 3).

The reduction in parasitemia levels was followed by protection against infection-induced weight loss. Notably, infected mice that received no treatment, especially those from the Swiss lineage, exhibited weight loss, mirroring the trend observed in the amiodarone-treated groups (Figure 4A,B). The ability of ravuconazole to protect the animals from weight loss was again dose dependent, while all mice treated with benznidazole and combination therapy gained weight similar to healthy mice (Figure 4A,B). Throughout the study, no mortality was observed except for two Swiss mice, one from the untreated group and one from the amiodarone-treated group, which succumbed on the 12th day postinfection. These mice exhibited the highest parasitemia levels within their respective groups (data not shown).

**Figure 4:**
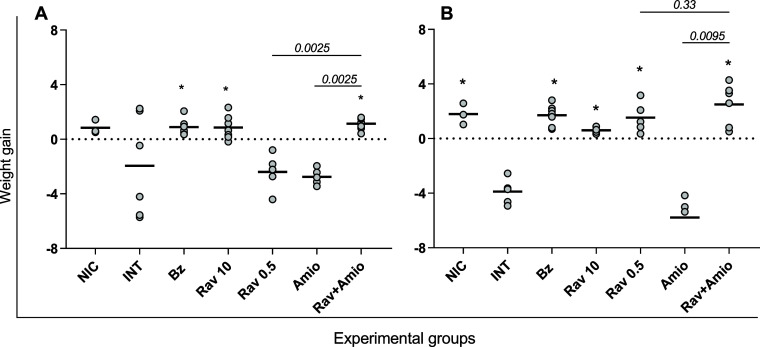
Combination therapy protects mice infected with Y T. cruzi strain from weight loss. Weight gain observed in BALB/c (A) and Swiss (B) mice infected with Y T. cruzi strain and treated with benznidazole at 100 mg/kg body weight (Bz); ravuconazole at 0.5 mg/kg (Rav 0.5); ravuconazole at 10 mg/kg (Rav 10); amiodarone at 50 mg/kg (Amio) or a combination of ravuconazole 0.5 mg/kg and amiodarone 50 mg/kg (Rav + Amio). Infected untreated controls (INT) received water. NIC—non infected controls. Treatments were administered orally from the 5th to the 9th day after infection. Weight gain was calculated as the difference between final weight (14 days postinfection) and initial weight (5 days postinfection). Data are presented as mean ± SD and analyzed using the Mann–Whitney test. Asterisks indicate significant differences compared to infected untreated mice. *p < 0.05. *p*-Values for comparisons between combination therapies and monotherapies at equivalent doses are shown in the graphs.

### Underdosed Ravuconazole Impacts IL-6 Levels in a Host-Dependent Manner, Whereas Drug Combination Restores Cytokine Levels in Infected BALB/c and Swiss Mice

To improve our understanding of the role of mouse lineage in immune responses and treatment outcomes, we investigate the cytokine profile at 14 days after infection, corresponding to 5 days after the treatment ended. T. cruzi infection induced a significant increase of IFN-γ, tumor necrosis factor-α (TNF-α), IL-6, and IL-10, in both mice lineages, while IL-2 and IL-4 levels were not altered (Figures 5 and 6).

**Figure 5:**
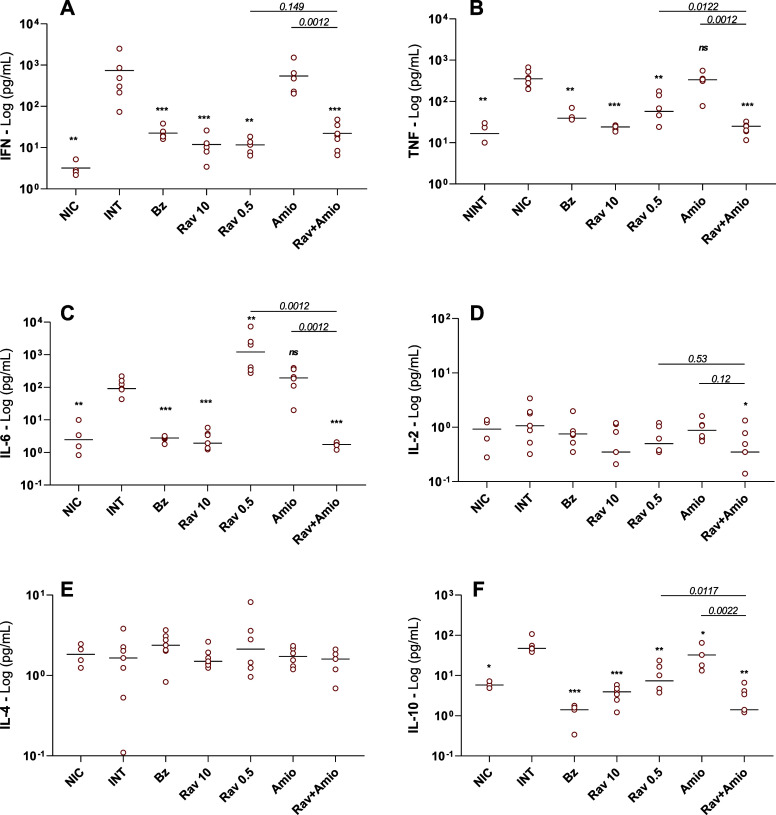
Cytokine profiles from BALB/c mice infected with Y T. cruzi strain and treated or untreated. BALB/c mice were infected with Y T. cruzi strain and treated with benznidazole at 100 mg/kg body weight (Bz); ravuconazole at 0.5 mg/kg (Rav 0.5); ravuconazole at 10 mg/kg (Rav 10); amiodarone at 50 mg/kg (Amio) or a combination of ravuconazole 0.5 mg/kg and amiodarone 50 mg/kg (Rav + Amio). Infected untreated controls (INT) received water. NIC—non infected controls. Treatments were administered orally from the 5th to the 9th day after infection. IFN-γ (A), TNF-α (B), IL-6 (C), IL-2 (D), IL-4 (E), and IL-10 (F) were quantified in plasma samples collected on the 14th day post-infection. Data are presented as mean ± SD. Asterisks indicate significant differences compared to infected untreated mice. *p < 0.05, **p < 0.01, ***p < 0.001. *p*-Values for comparisons between combination therapies and monotherapies at equivalent doses are shown in the graphs.

**Figure 6:**
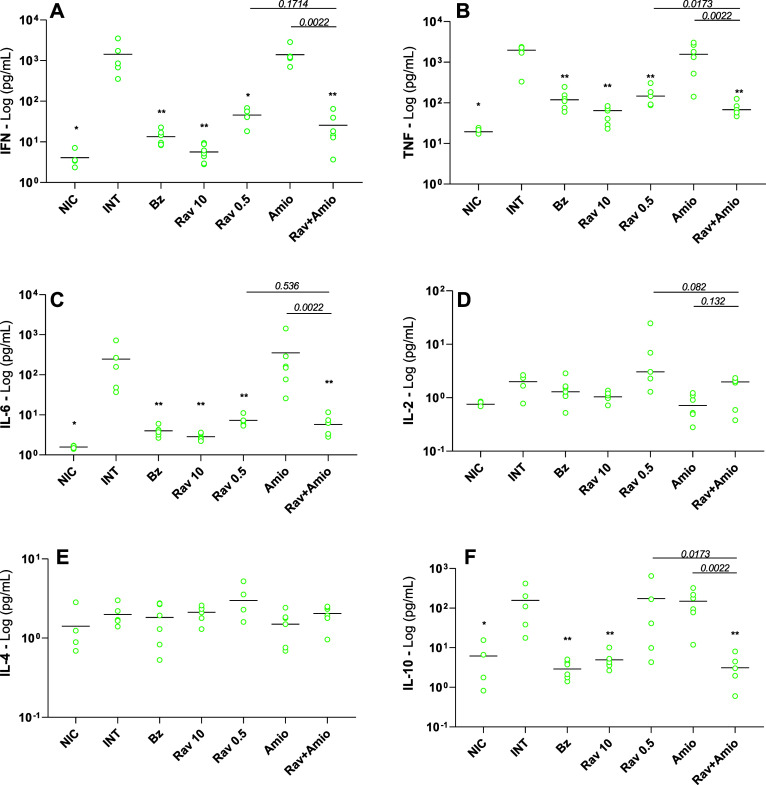
Impacts of acute T. cruzi infection and etiological treatments on cytokine profiles from Swiss mice. Swiss mice were infected with Y T. cruzi strain and treated with benznidazole at 100 mg/kg body weight (Bz); ravuconazole at 0.5 mg/kg (Rav 0.5); ravuconazole at 10 mg/kg (Rav 10); amiodarone at 50 mg/kg (Amio) or a combination of ravuconazole 0.5 mg/kg and amiodarone 50 mg/kg (Rav + Amio). Infected untreated controls (INT) received water. NIC—non infected controls. Treatments were administered orally from the 5th to the 9th day after infection. IFN-γ (A), TNF-α (B), IL-6 (C), IL-2 (D), IL-4 (E), and IL-10 (F) were quantified in plasma samples collected on the 14th day post-infection. Data are presented as mean ± SD. Asterisks indicate significant differences compared to infected untreated mice. *p < 0.05, **p < 0.01, ***p < 0.001. *p*-Values for comparisons between combination therapies and monotherapies at equivalent doses are shown in the graphs.

Treatment with benznidazole and ravuconazole at higher doses interfered with cytokine production, with the IFN-γ, TNF-α, IL-6, and IL-10 levels in these cases being significantly lower than those observed for infected and untreated mice. Conversely, amiodarone was not able to alter the cytokine profile observed in infected untreated mice, except for IL-10 in BALB/c mice (Figure 5F).

Regarding ravuconazole at 0.5 mg/kg, reduced IFN-γ and TNF-α levels were observed in all treated mice, but the effects on IL-6 and IL-10 were dependent on host lineage; the drug did not interfere with the IL-10 levels of infected Swiss mice (Figure 6F) but a significant decrease was observed for BALB/c animals (Figure 5F). The IL-6 profile, in comparison with infected and untreated mice, was antagonistic between the models, with a significant increase in BALB/c and the opposite in Swiss mice (Figures 5C and 6C). Remarkably, the effects of combination therapy on reducing cytokine levels compared with monotherapies with the same doses were observed for both lineages, particularly for TNF-α (Figures 5B and 6B) and IL-10 (Figures 5F and 6F). In BALB/c mice treated with a combined therapy, the IL-6 levels were also significantly reduced (Figure 5C).

### Treatments Impact Differentially on Parasite Burden in the Heart, Cardiac Tissue Damage, and the Splenic Index

To assess the impact of treatments on tissue lesions, we performed histopathological and morphometric analyses of the myocardium. Representative photomicrographs and cellular nuclei counts are shown in Figure 7. Uninfected mice displayed typical cardiomyocyte architecture (Figure 7E) while infected ones exhibited higher cellularity (Figure 7A,B) and amastigotes nests accompanied by moderate inflammatory infiltrate and signs of cellular degeneration, including vacuolization, increased acidophilia, loss of cellular boundaries, karyolysis, and connective tissue deposition (Figure 7E). In all infected mice treated with benznidazole and ravuconazole at higher doses, no signs of cellular degeneration or amastigotes nests were seen (Figure 7E). However, a small infiltration of mononuclear cells was present, with the nuclei count significantly lower than in untreated controls for both lineages (Figure 7A,B). In contrast, while no nests of amastigotes were observed in mice treated with 0.5 mg/kg ravuconazole (both BALB/c and Swiss strains), alterations similar to or worse than those seen in untreated mice were present, especially in BALB/c mice (Figure 7E). In animals treated with amiodarone, for both mouse lineages, the mean number of nuclei in the myocardium of amiodarone-treated mice was significantly lower than that in infected controls (Figure 7A,B). However, numerous amastigotes nests and extensive areas of cellular degeneration and replacement by connective tissue were identified (Figure 7E). Conversely, tissues from Swiss and BALB/c mice treated with drug combination showed scant mononuclear cells, with no signs of cellular degeneration or amastigotes nests (Figure 7E).

**Figure 7:**
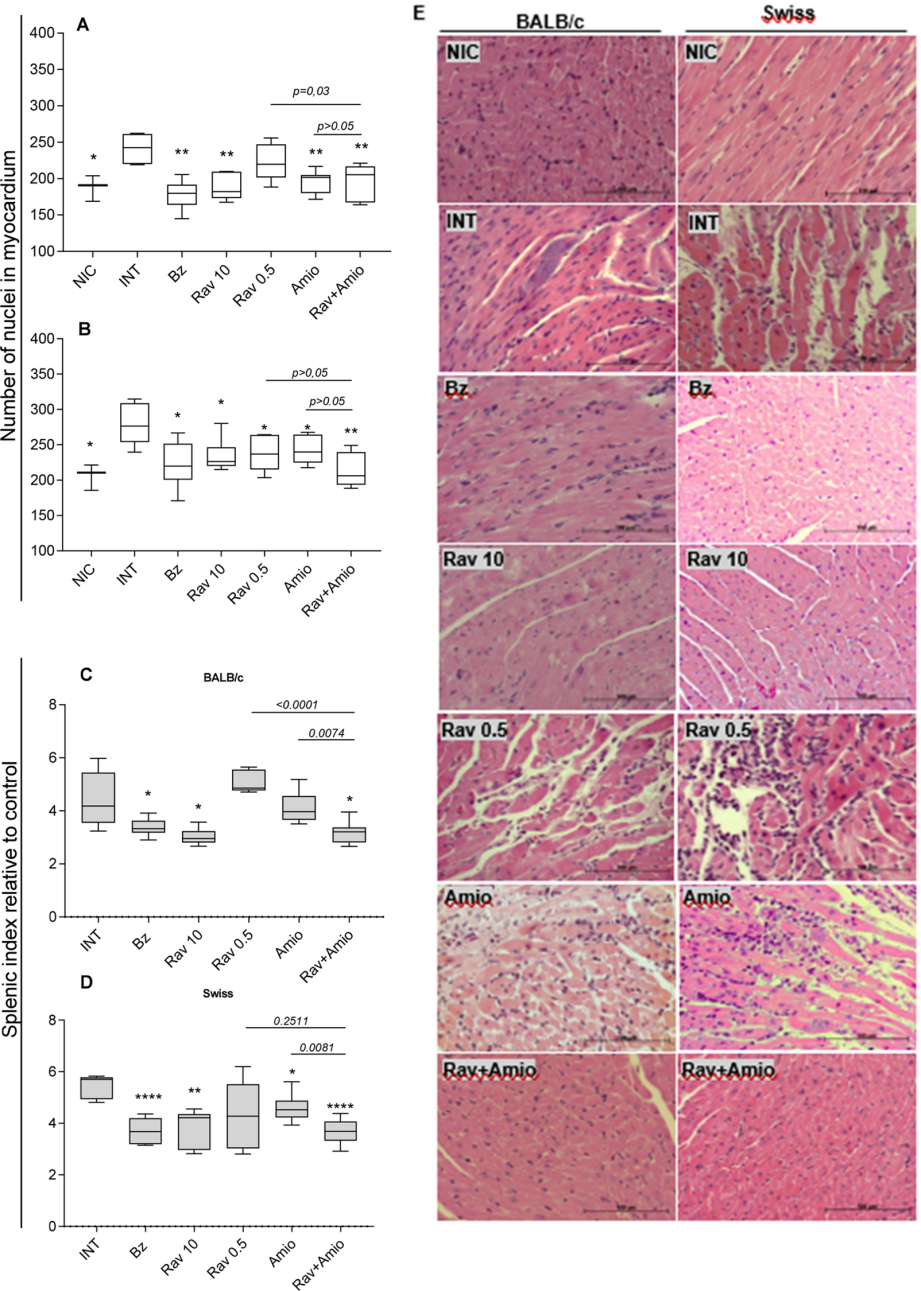
Effects of T. cruzi infection and etiological treatments on cardiac tissue and splenic index in BALB/c and Swiss mice. Number of nuclei measured in the myocardium of (A) BALB/c and (B) Swiss mice; (C) splenic index from BALB/c and (D) Swiss mice; (E) representative photomicrographs (hematoxylin and eosin staining) of hearts from BALB/c (left panel) and Swiss (right panel) mice infected with Y T. cruzi strain and treated with benznidazole at 100 mg/kg body weight (Bz); ravuconazole at 0.5 mg/kg (Rav 0.5); ravuconazole at 10 mg/kg (Rav 10); amiodarone at 50 mg/kg (Amio) or a combination of ravuconazole 0.5 mg/kg and amiodarone 50 mg/kg (Rav + Amio). Infected untreated controls (INT) received water. NIC—non infected controls. Treatments were administered orally from the 5th to the 9th day after infection. Hearts and spleens were collected on the 14th day post-infection. The splenic index was calculated as the spleen weight divided by the body weight × 100 for each animal and normalized to the mean index of uninfected mice. Asterisks indicate significant differences compared to infected, untreated mice. *p < 0.05, **p < 0.01; ****p < 0.0001. *p*-Values for comparisons between combination therapies and monotherapies at equivalent doses are shown in the graphs.

In addition to assessing myocarditis, we evaluated the splenic index as an indirect measure of the systemic immune response associated with an acute infection. Figure 7C,D shows that all infected mice had relative spleen mass at least twice that of uninfected ones at 14th of infection. However, in the treated mice in which parasitemia was reduced or suppressed, benznidazole, ravuconazole 10 mg/kg, and drug combination, the splenic index was significantly reduced in comparison with untreated animals. A slight reduction was also observed in Swiss mice treated with amiodarone (Figure 7D), despite high parasitemia levels (Figure 3B,D). The positive effect resulting from combination therapy was clearly noticed for BALB/c lineage, where the spleen mass was significantly lower in combination treated mice compared with monotherapies at the same doses (Figure 7C) mirroring the effect observed with parasitemia (Figure 3A,C).

### Ravuconazole and Amiodarone in Combination Reduce Parasitemia and Splenic Index of BALB/c Infected with a Benznidazole-Resistant Strain

Considering the promising results obtained treating a partially resistant strain, in the next step, we evaluated the effects of ravuconazole and amiodarone combination against the highly drug-resistant Colombian strain. Using the BALB/c model in a short-course treatment, we investigated the parasitemia, body weight, cytokine profile, and cardiac lesions resulting from monotherapies with amiodarone at 50 mg/kg, ravuconazole at lower dose (0.5 mg/kg), and combinations at these same doses. As shown in Figure 8A,B, amiodarone did not result in any anti-T. cruzi activity since the parasitemia was similar to infected nontreated mice in all of the periods evaluated, as well as the loss weight induced by infection in 50% untreated mice and amiodarone-treated ones (Figure 8C). Conversely, ravuconazole alone and in combination significantly reduced parasitemia (Figure 8A), resulting in an AUC lower than that of amiodarone-treated and nontreated infected mice (Figure 8B). However, after the treatments ended, mice that received combination therapy retained low levels of parasites, while ravuconazole as a monotherapy showed a clear tendency toward increasing parasitemia, particularly on days 18 and 19 postinfection (Figure 8A).

**Figure 8:**
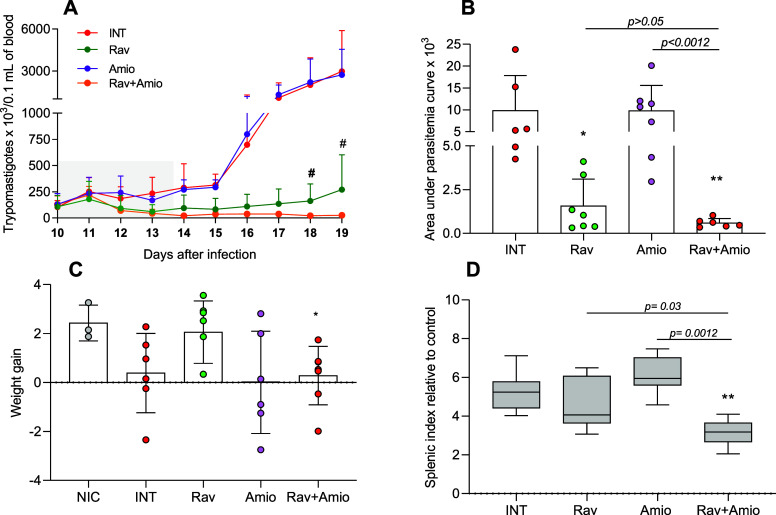
In vivo impacts of ravuconazole and/or amiodarone in monotherapy and combination against acute T. cruzi infection by the Colombian strain. (A) Parasitemia curve, (B) area under parasitemia curve, (C) weight gain, and (D) splenic index of BALB/c mice infected with Colombian T. cruzi strain and treated with ravuconazole at 0.5 mg/kg body weight (Rav 0.5); amiodarone (Amio) at 50 mg/kg and a combination of ravuconazole 0.5 mg/kg and amiodarone 50 mg/kg (Rav + Amio). INT—untreated infected control. NIC—noninfected control. The treatments were administered orally from the 10th to the 14th day after infection. Weight gain as calculated as the difference between final weight (19 days postinfection) and initial weight (10 days postinfection). The splenic index was determined on the 19th day post-infection and calculated as the spleen weight divided by the body weight × 100 for each animal and normalized to the mean index of uninfected mice. (A–C) Mean ± SD, (D) horizontal lines represent the median. # indicates significant difference comparing ravuconazole and combination group (*p* < 0.05). Asterisks indicate significant differences compared to infected untreated mice. *p < 0.05, **p < 0.01. *p*-Values for comparisons between combined therapy and monotherapies at equivalent doses are shown in the graphs.

Consistent with these findings, the splenic index of animals treated with the drug combination was significantly lower than those of those treated with monotherapies at the same doses or infected untreated controls (Figure 8D). The treatment with ravuconazole in monotherapy protected mice from weight loss; however, despite lower parasitemia, 28.5% of mice treated with the drug combination experienced weight loss and their mean weight gain was significantly lower than that of uninfected controls (Figure 8C).

### Combined Therapy Restores the Balance between Pro and Anti-inflammatory Cytokines While Reducing Parasite Load and Cardiac Inflammation in Mice Infected with the Colombian Strain

Figure 9 shows the serum cytokine levels on the last day of the experiment, i.e., 19 days postinfection by the Colombian T. cruzi strain. A significant increase was detected in the IFN-γ, TNF-α, and IL-6 levels in infected untreated mice (Figure 9A–C); a similar profile was observed for treatments with ravuconazole and amiodarone in monotherapies. Interestingly, mice treated with the drugs in combination showed reduced levels of IFN-γ and TNF-α in comparison to untreated mice. We did not observe any changes in IL-2, IL-4, or IL-10 levels resulting from the infection (Figure 9D–F); however, ravuconazole-treated mice presented higher levels of IL-4, and combination therapy significantly increased the IL-4 and IL-10 compared to untreated infected mice.

**Figure 9:**
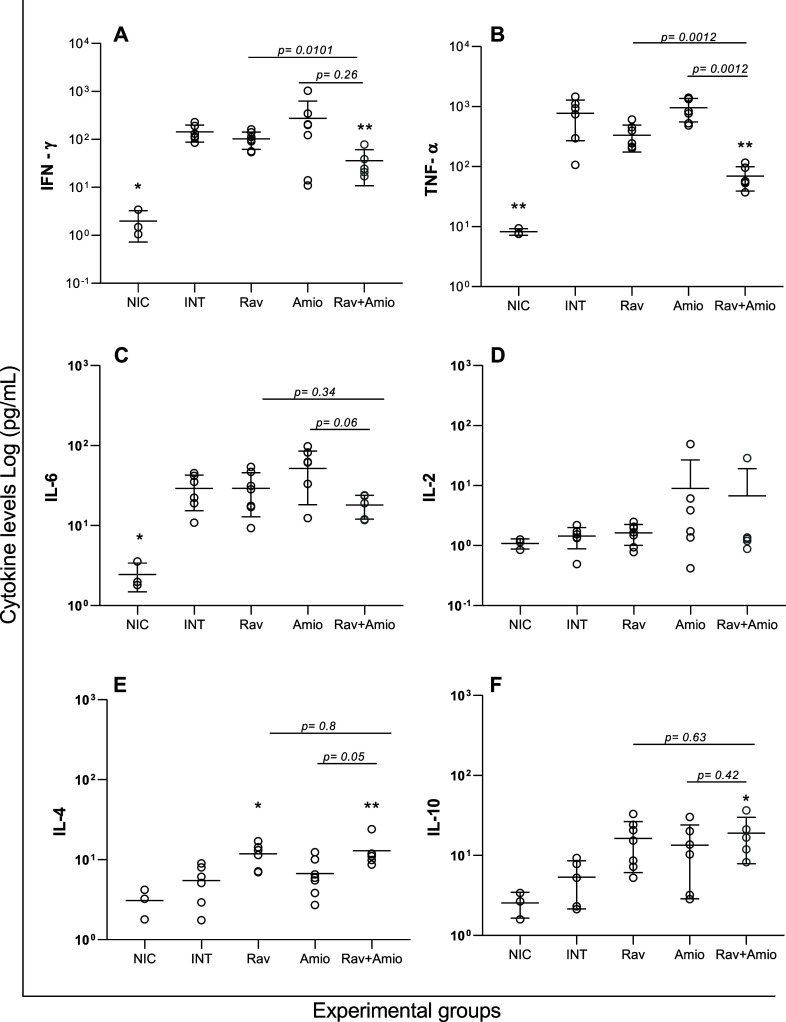
Ravuconazole and amiodarone in combination impacts on the cytokine profile of BALB/c mice infected with Colombian T. cruzi strain. Pro and anti-inflammatory cytokines detected in sera from BALB/c mice infected with Colombian T. cruzi strain and treated with ravuconazole at 0.5 mg/kg body weight (Rav 0.5); amiodarone at 50 mg/kg (Amio) or a combination of ravuconazole 0.5 mg/kg and amiodarone 50 mg/kg (Rav + Amio). Infected untreated controls (INT) received water. NIC—non infected controls. Treatments were administered orally from the 10th to the 14th day after infection. IFN-γ (A), TNF-α (B), IL-6 (C), IL-2 (D), IL-4 (E), and IL-10 (F) were quantified in plasma samples collected on the 19th day post-infection. Data are presented as mean ± SD. Asterisks indicate significant differences compared to infected, untreated mice. *p < 0.05, **p < 0.01. *p*-Values for comparisons between combination therapies and monotherapies at equivalent doses are shown in the graphs.

The quantification of myocardial nuclei revealed that infected animals, regardless of treatment status, exhibited a higher number of nuclei compared to the uninfected control group (Figure 10A). In untreated mice, numerous amastigote nests, extensive infiltration of inflammatory cells, degenerating cardiomyocytes, and large areas of connective tissue deposition were observed (Figure 10B). Similarly, all amiodarone-treated animals displayed multiple inflammatory infiltrates and frequently parasitized cells, closely resembling the infected control group (Figure 10B). In the group treated with ravuconazole, 3 out of 7 mice showed no evidence of amastigotes nests or relevant signs of cellular injury, while the remaining animals exhibited histopathological features similar to those of the infected untreated group (Figure 10A,B). In contrast, the cardiac tissue of animals treated with the combination therapy showed rare amastigote nests and, when present, only sparse inflammatory infiltrates with well-preserved tissue architecture (Figure 10E).

**Figure 10:**
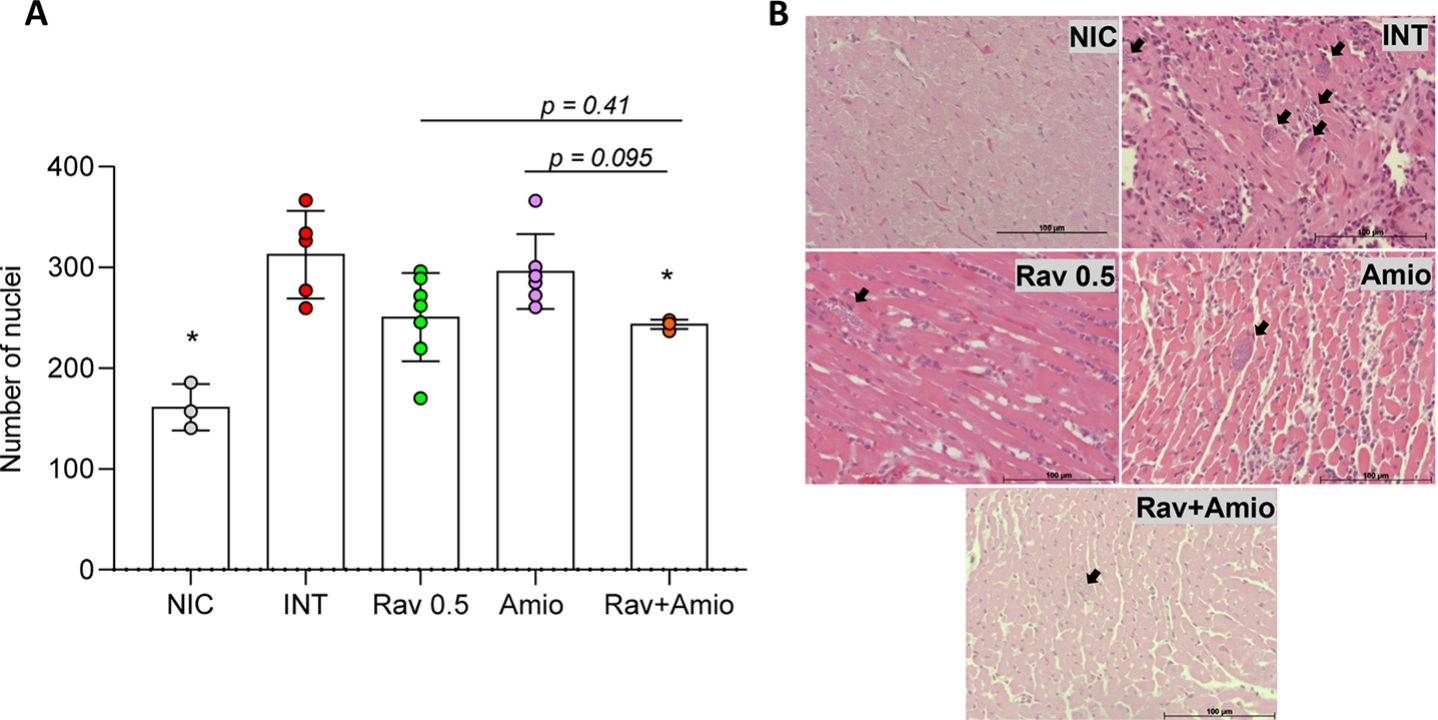
The combination of ravuconazole and amiodarone reduces heart parasite load and myocarditis in mice infected with a benznidazole-resistant strain. (A) Number of nuclei measured in myocardium and (B) representative photomicrographs (hematoxylin and eosin staining) of hearts from BALB/c mice infected with Colombian T. cruzi strain and treated with ravuconazole at 0.5 mg/kg body weight (Rav 0.5); amiodarone at 50 mg/kg (Amio) or a combination of ravuconazole 0.5 mg/kg and amiodarone 50 mg/kg (Rav + Amio). Infected untreated controls (INT) received water. NIC—non infected controls. Treatments were administered orally from the 10th to the 14th day after infection. Hearts were collected on the 19th day post-infection. Data are presented as mean ± SD. Asterisks indicate significant differences compared to infected, untreated mice. *p < 0.05. *p*-Values for comparisons between combination therapies and monotherapies at equivalent doses are shown in the graph. Arrows indicate amastigote nests.

## Discussion

Although the development of new drugs with novel mechanisms of action anti-T. cruzi remains a priority, comprehensive exploration of approved drugs and validated drug candidates may provide valuable insights and contribute to the further discovery of newer chemotherapeutic options.^9,27^ Furthermore, combination therapy using repurposed drugs presents a promising approach to accelerate drug development.^27^ However, the limited understanding of experimental models for testing drug combinations poses a significant barrier to advancing these therapies. Generating robust data on strategies to identify the most promising combinations and prioritizing their evaluation are essential steps toward improving chemotherapy for Chagas disease.

Previous reports have shown that amiodarone can synergize with azoles against fungi^24,25^ and T. cruzi.^18,22,26^ Here, we investigated whether amiodarone enhances the trypanocidal effect of ravuconazole, a triazole derivative, against parasite strains with partial or complete resistance to the reference drugs. Additionally, we assessed the potential influence of the host model on therapeutic outcomes. For this purpose, we selected inbred BALB/c and outbred Swiss mice, considering that much of our understanding of experimental chemotherapy anti-T. cruzi is based on studies conducted using these lineages.^17,28^

Contrary to previous data demonstrating potential to treat experimental Chagas disease,^21,22,29^ we did not observe any anti-T. cruzi in vivo activity of amiodarone at 50 mg/kg in acute models using Y or Colombian strains. Similarly, Francisco et al.^30^ reported no trypanocidal effects from oral administration of amiodarone and argued that its systemic exposure is insufficient to reach the anti-T. cruzi IC_50_.^30^ Thus, amiodarone monotherapy at approved human doses is unlikely to be effective for the treatment of Chagas disease treatment.

Interestingly, despite its lack of activity as a monotherapy, amiodarone demonstrated a clear in vivo interaction with ravuconazole, regardless of the host or parasite strain. Combined treatment with an underdosed ravuconazole (0.5 mg/kg) achieved parasite load reductions comparable to those obtained with the optimal ravuconazole dose (10 mg/kg). This combination effectively suppressed parasitemia in Y strain-infected mice and significantly reduced parasitemia in Colombian strain-infected mice, outperforming monotherapies at the same doses. These findings align with previous studies demonstrating positive interactions between amiodarone and posaconazole and itraconazole against T. cruzi strains susceptible or partially resistant to benznidazole.^19,22,26^ Collectively, these data underscore the importance of a detailed investigation into the pharmacokinetic/pharmacodynamic properties of this drug combination.

Amiodarone is known to participate in numerous drug interactions by inhibiting CYP3A4 and P-glycoprotein (PgP).^31,32^ While data on ravuconazole’s metabolism is limited, it is likely metabolized by CYP3A4 ^33^ and its prodrug, E1224, shows lower potential for drug–drug interactions compared to other azoles.^34^ Given these findings, it is reasonable to hypothesize that amiodarone may enhance ravuconazole’s activity by slowing its metabolism and inhibiting PgP efflux, potentially improving absorption.^35^ Benaim et al.^22^ suggested that synergistic effects of posaconazole and amiodarone on T. cruzi result from two mechanisms—disruption of the parasite’s Ca^2+^ homeostasis and blockade of ergosterol biosynthesis. In fungi, amiodarone and fluconazole synergize by disrupting ergosterol synthesis and compensatory pathways, indicating similar mechanisms may apply to T. cruzi.^25^ Additionally, amiodarone induces global disruption of cell cycle progression and nutrient response in fungi,^36^ which may also affect T. cruzi. Further studies are needed to confirm whether amiodarone enhances ravuconazole bioavailability or interacts via other mechanisms in the treatment of T. cruzi infection. These studies should also assess potential toxicity or side effects as significant weight loss was observed in Colombian-infected mice treated with amiodarone and ravuconazole in combination, despite no toxicity from the monotherapies.

To comprehensively evaluate treatment outcomes beyond the antiparasitic effects, we assessed the immune response and cardiac damage associated with T. cruzi infection and therapies. There is evidence that the biological heterogeneity of T. cruzi affects tissue distribution and resistance to etiological treatment.^37^ Furthermore, mouse lineage plays a significant role in shaping the immune response to T. cruzi infection.^37–40^ However, there is limited information about how these factors interact to impact treatment outcomes.

We observed that acute infection by the Y strain induced a strong systemic proinflammatory Th1 response in both mouse lineages accompanied by elevated IL-10 levels. A similar immune profile was noted for the Colombian strain in BALB/c mice, except for lower IL-10 levels. IFN-γ and TNF-α are central players in controlling T. cruzi by activating macrophage and promoting nitric oxide production^41^ and can synergize with IL-6.^42^ This process is accompanied by releasing inflammatory products that are potentially deleterious to the host. IL-10, however, modulates the inflammation intensity, mitigating tissue damage.^42,43^ Our histopathologic analysis revealed less myocarditis in Y strain-infected strains compared to those infected with the Colombian strain. This suggests that the simultaneous rise of pro-inflammatory cytokines and IL-10 may help protect tissue in Y strain infections. Furthermore, the tissue tropism of T. cruzi strains is a crucial factor to consider. As observed by Reis Machado et al., 2014,^44^ mice infected with the Colombian strain, known for its myotropic nature,^45^ exhibited a higher frequency of amastigote nests in the myocardium. These findings highlight the importance of studying immune responses in experimental models as the distinct outcomes of human T. cruzi infections are also linked to immune profiles.^42,43^ While efforts to enhance the translational value of experimental models are ongoing,^16,17^ data on immune responses to commonly used parasite strains in treatment efficacy studies remain limited.

Our findings also highlighted the influence of host genetic background and parasite strain on drug activity, particularly at a low ravuconazole dose (0.5 mg/kg). In the Y strain infected mice, reductions in IFN-γ and TNF-α levels were observed across both mouse lineages. However, in the BALB/c mice, the worst drug performance was linked to a marked increase in IL-6 levels and a reduction in IL-10, suggesting an imbalance between proinflammatory and anti-inflammatory mediators. This imbalance may help explain the more severe inflammation and tissue damage in BALB/c mice, despite low cardiac parasite load, compared to Swiss mice.^38,39^ Conversely, in Colombian strain-infected mice, ravuconazole reduced parasitemia during treatment but cytokine levels and cardiac injury were comparable to untreated controls, except for IL-4. Amastigote nests were frequently observed in the myocardium, consistent with the reduced efficacy of ravuconazole against the Colombian strain in prior long-term studies in murine models.^18^

Amiodarone treatment in BALB/c and Swiss mice infected with the Y strain resulted in cardiac parasite loads similar to those of untreated controls but reduced myocardial cellularity, despite elevated proinflammatory cytokines. Previous studies have suggested amiodarone’s anti-inflammatory effects in chronic Chagas disease patients^46^ and experimental models of acute^29^ and chronic infection.^47^ However, under our experimental conditions, the reduced myocardial cellularity was accompanied by extensive cellular degeneration, similar to that in untreated mice. Additionally, amiodarone did not reduce inflammation or heart damage in BALB/c mice infected with the Colombian strain. This suggests that the ability of amiodarone to reduce myocardial cell infiltration may occur only at low parasitic loads and is insufficient to prevent cellular damage caused by acute infection.

In contrast, all treatments that effectively reduced parasitemia, such as combination therapy and reference regimens, were equally effective in modulating cytokine levels and reducing splenomegaly. These findings align with previous research indicating that transient suppression of parasite load through etiological treatment can shift immune profiles in both experimental T. cruzi infection^13,23,29,47^ and human Chagas disease.^48^ At all, this evidence indicates that although parasitemia is an important marker of effect in short-term protocols, other parameters, such as the cytokine profile and tissue histology, may help to predict the host drug response, even in the early acute phase.

Of the many translational challenges facing Chagas disease drug development,^16,17^ those related to combination therapy have been scarcely discussed. Although several drug combinations, particularly involving repurposed drugs, have been tested on experimental models, how best to combine the drugs to get long-lasting anti-T. cruzi response remains a critical question. The design of in vivo studies is complicated by the vast number of potential drug and dose combinations, coupled with the long time and complex protocols required to confirm parasitological cure post treatment.^16,17^ These limitations restrict the testing of multiple dose combinations. Therefore, innovative drug combination screening strategies are needed to identify promising candidates and guide comprehensive long-term preclinical evaluations.

This study used a short-term protocol, optimized from previous studies by our group,^5,13^ to investigate drug interactions in vivo, focusing on both parasite and host effects. The approach revealed that very low doses of ravuconazole, but no optimal doses, enabled detection of strain- and host-dependent responses as well as drug interactions that might be overlooked with higher doses. Notably, the response in BALB/c mice aligned with results from previous long-term studies using the same parasite strains.^13,18^ Based on these findings, we suggest that BALB/c mice infected with the Colombian strain constitute a suitable model for preclinical drug combination screening in short-term protocols, facilitating the selection of promising combinations for further long-term efficacy assessments.

Our data raise the issue of how in vitro results can be translated to in vivo models. In our in vitro studies, amiodarone demonstrated trypanocidal activity associated with significant toxicity to host cells with a low selectivity index (∼1.4). When combined with ravuconazole, this toxicity appeared to be slightly attenuated and the combination showed an additive anti-T. cruzi effect. Recognizing the potential for noncytotoxic additive and synergistic drug combinations, we advanced the study of ravuconazole and amiodarone combination to murine models of Chagas disease. Furthermore, preclinical^12,13,19^ and clinical data^49^ suggest that combinations with merely additive effects in vitro can exhibit enhanced interactions in vivo. These findings underscore the importance of establishing parameters to guide the decision-making process for advancing anti-T. cruzi drug combinations from in vitro to in vivo evaluations.

Overall, we found a consistent protective effect from the combination of ravuconazole and amiodarone. Further investigations are needed to unravel the potential of this combination in promoting long-lasting anti-T. cruzi activity. Additionally, our results highlight the impact of both mouse genetic background and parasite strain on host responses to treatments, which should be considered when evaluating new drug candidates for Chagas disease. Finally, we present short-term in vivo models for screening drug combinations, enabling a comprehensive assessment of their effects on parasite load, immune response, and heart damage.

## Methods

### Drugs

Ravuconazole was purchased from Sigma-Aldrich (St. Louis, MO, USA). Amiodarone hydrochloride was purchased from Sanofi Aventis (Paris, France). Benznidazole, purchased from LAFEPE (Pernambuco State Pharmaceutical Laboratory), was used as the reference treatment.

For in vitro studies, stock solutions of ravuconazole (10 mM), amiodarone (25 mM) and benznidazole (100 mM) were prepared in pure dimethyl sulfoxide (DMSO-Merck). The dilutions of each drug, as a monotherapy or in combination, were prepared in fresh culture medium on the day of the assay. For in vivo studies, amiodarone and benznidazole were administered in aqueous suspensions containing 0.5% w/v carboxymethyl cellulose, and ravuconazole was solubilized in DMSO and diluted with water. Maximum volumes of 0.25 and 5.0 μL of DMSO were administered to mice treated with ravuconazole at doses of 0.5 and 10 mg/kg body weight, respectively.

### Parasites and Mammalian Cell Cultures

Trypanosoma (Schizotrypanum) cruzi Colombian and Y and strains representative of genotype discrete typing units I and II, respectively, were employed. The strains exhibit different patterns of resistance to reference treatment; Y strain is partially resistant to benznidazole and Colombian is fully resistant to benznidazole.^17^

The embryonic rat ventricular cell line H9c2 was used for both drug toxicity and infection assays. The cultures were maintained in Dulbecco’s modified Eagle’s medium supplemented with 10% fetal bovine serum, 1 mM l-glutamine, and 100 μg/mL penicillin/streptomycin. Cell cultures were maintained at 37 °C in an atmosphere with a 5% CO_2_/air mixture. All assays were independently conducted at least twice.

### Cell Toxicity Assessment

Noninfected H9c2 cells were cultured at 37 °C for 48 h with increasing concentrations of ravuconazole (6.25 nM up to 100 nM) and amiodarone (12.5 μM up to 200 μM). Cell viability was assessed using a spectrophotometer through the resazurin assay.^13^ The results were expressed as the difference in resazurin reduction between cells incubated with the drugs and those without. The toxicity of the combinations was evaluated by combining the highest concentrations of ravuconazole and amiodarone, followed by five points of 1:2 serial dilutions.

### Anti-T. cruzi Effect

The effect of ravuconazole and amiodarone in monotherapy and combination in vitro was verified on T. cruzi amastigotes, as previously described.^13^ Briefly, the assays were carried out using H9c2 cells infected with trypomastigotes obtained from a tissue culture of the Y strain. Top concentrations of ravuconazole (10 nM) and amiodarone (20 μM), in monotherapy and combined in a 1:1 rate followed by five 1:2 dilutions, were incubated with infected cells. The plates were maintained at 37 °C in a 5% CO_2_/air mixture. After 48 h, the cultures were fixed with methanol, stained with Giemsa, and microscopically examined to determine the percentage of cells infected in the absence and presence of the drugs. These data were used to calculate the 50% and 90% effective concentrations—EC_50_ and EC_90_, and the fractional inhibitory concentrations at EC_50_ and EC_90_ levels—FIC_50_ and FIC_90_ using the software Compusyn (ComboSyn., Paramus, USA). The ∑FIC was used to classify the nature of the interaction.^17^ All experiments were run in duplicate, and the results were presented as the means ± standard deviation from at least two independent experiments.

### In Vivo Studies

#### Mouse Infection and Ethics

Female Swiss and BALB/c mice (18–20 g) were acquired from the Animal Facility at Universidade Federal de Minas Gerais (Belo Horizonte, Minas Gerais State, Brazil) and were housed in a standard room maintained at a temperature of 20–24 °C under a 12/12 h light/dark cycle. The animals had access to commercial feed and drinking water ad libitum. Each group of animals (5 to 7 per group) was intraperitoneally inoculated with 5.0 × 10^3^ bloodstream trypomastigotes of the Y T. cruzi or Colombian strain. The parasites were sourced from the blood of previously infected animals. Control groups of uninfected animals (3 to 4 per group) were also included in the assays. It is worth mentioning that all experimental procedures were carried out at the same time for the two mice lineages infected with the Y strain, with parasites and drugs derived from the same stocks being used, enabling the comparison. All procedures were approved by the Institutional Ethics Committee for Animal Research (Protocol number 04/2019).

#### Treatments

After parasitemia detection (5 days postinoculation for the Y strain and 10 days for the Colombian strain), the drugs were administered by gavage for 5 consecutive days. Infected mice were treated with ravuconazole (Rav) at 0.5 mg/kg body weight^13^ and amiodarone (Amio) at 50 mg/kg^21,28^ in monotherapy. Animals receiving combined chemotherapy (Rav + Amio) were administered ravuconazole (0.5 mg/kg) and amiodarone (50 mg/kg), with a 30 min interval between the administrations to minimize potential gastrointestinal interactions and to allow adequate absorption of the first drug, ensuring more predictable pharmacokinetic profiles. Infected and untreated control mice (INT) received water by gavage in the same volume used for drug administration. For experiments involving the Y strain, additional treatments included the reference drug benznidazole (100 mg/kg)^5^ and ravuconazole at 10 mg/kg.^13^

#### Data Collection

Parasitemia, body weight, and mortality were monitored from the onset of infection until 5 days post-treatment. Mice were then euthanized, and blood, spleen, and heart were collected, corresponding to 14 days postinfection for Y strain and 19 days postinfection for Colombian strain-infected mice.

#### Fresh Blood Examination

Parasitemia was determined by a fresh blood examination. Five microliters of blood collected from the tail vein were examined and the parasite number was estimated as described previously.^13^

#### Weight Gain and Splenic Index

Weight gain was calculated as the difference between the final weight (on the last day of the experiment) and the initial weight (on the day that treatment began).

The splenic index was calculated as the spleen weight divided by the body weight × 100 for each animal and normalized to the mean index of uninfected mice.

#### Heart Histology

Cardiac fragments were collected, stored in 10% buffered formalin solution for 48 h, then dehydrated in ethanol. The fragments were diaphanized in xylene and embedded in paraffin. Serial sections (4 μm thick) were obtained by using a microtome. These sections were mounted on glass slides and stained with hematoxylin and eosin. Twenty random, nonoverlapping histological fields representing all myocardial regions were analyzed. The images were observed under a 40× objective lens (400× magnification) and scanned using an Axioscope A1 photomicroscope coupled with the AxioVision image analysis software (Carl Zeiss, Germany). Cellular nuclei were counted in each image to assess the presence of myocarditis, while histopathological alterations and the presence of amastigotes nests were also recorded.

#### Cytokine Circulating Levels

Cytokine levels were evaluated in plasma samples collected 5 d after the end of the treatments. Interleukin (IL)-2, IL-4, IL-6, IL-10 and IL-17A, interferon γ (IFN-γ) and TNF-α, were measured by flow cytometry using a BD Cytometric Bead Array (CBA) Mouse Th1/Th2/Th17 Cytokine Kit (BD Biosciences, United States), according to the manufacturer’s instructions (BD Biosciences, San Jose, CA, USA).^13^ Samples were acquired in a BD FACSVerse flow cytometer, and data analyses were performed using CBA analysis FCAP Array software (BD Biosciences, San Jose, CA, USA).

### Statistical Analysis

All results were expressed as the mean ± standard deviation. Normality in the data distribution was checked with a Shapiro–Wilk test. Parametric data were analyzed with Student’s *t*-test and nonparametric data with the Mann–Whitney test using GraphPad Prism 8 (California, USA). The differences were considered significant if the *P* value was less than or equal to 0.05.

## References

[1] Müller
Kratz J., Garcia Bournissen F., Forsyth C. J., Sosa-Estani S. (2018). Clinical and
pharmacological profile of benznidazole for treatment of Chagas disease. Expet Rev. Clin. Pharmacol..

[2] Losada
Galván I., Alonso-Padilla J., Cortés-Serra N., Alonso-Vega C., Gascón J., Pinazo M. J. (2021). Benznidazole for
the treatment of Chagas disease. Expert Rev.
Anti Infect. Ther..

[3] Gómez-Ochoa S. A., Rojas L. Z., Echeverría L.
E., Muka T., Franco O. H. (2022). Global, Regional, and National Trends of Chagas Disease
from 1990 to 2019: Comprehensive Analysis of the Global Burden of
Disease Study. Glob. Heart.

[4] Soeiro M. N. C. (2022). Perspectives
for a new drug candidate for Chagas disease therapy. Mem. Inst. Oswaldo Cruz.

[5] Mazzeti A. L., Capelari-Oliveira P., Bahia M. T., Mosqueira V. C. F. (2021). Review
on Experimental Treatment Strategies Against *Trypanosoma cruzi*. J. Exp. Pharmacol..

[6] Morillo C. A., Waskin H., Sosa-Estani S., Del Carmen Bangher M., Cuneo C., Milesi R., Mallagray M., Apt W., Beloscar J., Gascon J., Molina I., Echeverria L. E., Colombo H., Perez-Molina J. A., Wyss F., Meeks B., Bonilla L. R., Gao P., Wei B., McCarthy M., Yusuf S., Morillo C. (2017). Benznidazole and Posaconazole
in Eliminating Parasites in Asymptomatic T. Cruzi Carriers: The STOP-CHAGAS
Trial. J. Am. Coll. Cardiol..

[7] Torrico F., Gascon J., Ortiz L., Alonso-Vega C., Pinazo M. J., Schijman A., Almeida I. C., Alves F., Strub-Wourgaft N., Ribeiro I., Santina G. (2018). Treatment
of adult chronic indeterminate Chagas disease with benznidazole and
three E1224 dosing regimens: a proof-of-concept, randomised, placebo-controlled
trial. Lancet Infect. Dis..

[8] Pinazo M. J., Forsyth C., Losada I., Esteban E. T., García-Rodríguez M., Villegas M. L., Molina I., Crespillo-Andújar C., Gállego M., Ballart C., Ramirez J. C., Aden T., Hoerauf A., Pfarr K., Vaillant M., Marques T., Fernandes J., Blum B., Ribeiro I., Sosa-Estani S., Barreira F., Gascón J., FEXI-12 Study Team (2024). Efficacy and safety of fexinidazole
for treatment of chronic indeterminate Chagas disease (FEXI-12): a
multicentre, randomised, double-blind, phase 2 trial. Lancet Infect. Dis..

[9] Porta E. O. J., Kalesh K., Steel P. G. (2023). Navigating drug
repurposing for Chagas
disease: advances, challenges, and opportunities. Front. Pharmacol.

[10] Saraiva R. M., Portela L. F., Silveira G. P. E., Gomes N. L. S., Pinto D. P., Silva A. C. A., Sangenis L. H. C., Carneiro F. M., Almeida-Silva J., Marinho P. W., Sperandio-Silva G. M., Estrela R. C. E., Hasslocher-Moreno A.
M., Mediano M. F. F., Moreira O., Britto C., Perez S. A. C., Viçosa A. L., Suarez-Fontes A. M., Vannier-Santos M. A. (2021). Disulfiram repurposing in the combined
chemotherapy
of Chagas disease: A protocol for phase I/II clinical trial. Med. Case Rep. Stud. Protoc..

[11] Torrico F., Gascón J., Barreira F., Blum B., Almeida I. C., Alonso-Vega C., Barboza T., Bilbe G., Correia E., Garcia W., Ortiz L., Parrado R., Ramirez J. C., Ribeiro I., Strub-Wourgaft N., Vaillant M., Sosa-Estani S., BENDITA Study Group (2021). New
regimens of benznidazole monotherapy and in combination with fosravuconazole
for treatment of Chagas disease (BENDITA): a phase 2, double-blind,
randomised trial. Lancet Infect. Dis..

[12] Pandey R. P., Nascimento M. S., Franco C. H., Bortoluci K., Silva M. N., Zingales B., Gibaldi D., Castaño
Barrios L., Lannes-Vieira J., Cariste L. M., Vasconcelos J. R., Moraes C. B., Freitas-Junior L. H., Kalil J., Alcântara L., Cunha-Neto E. (2022). Drug Repurposing in Chagas Disease: Chloroquine Potentiates
Benznidazole Activity against Trypanosoma cruzi In Vitro and In Vivo. Antimicrob. Agents Chemother..

[13] Machado Y. A., Bahia M. T., Caldas I. S., Mazzeti A. L., Novaes R. D., Vilas Boas B. R., Santos L. J. S., Martins-Filho O. A., Marques M. J., Diniz L. F. (2020). Amlodipine Increases the Therapeutic
Potential of Ravuconazole upon Trypanosoma cruzi Infection. Antimicrob. Agents Chemother..

[14] González S., Wall R. J., Thomas J., Braillard S., Brunori G., Camino Díaz I., Cantizani J., Carvalho S., Castañeda Casado P., Chatelain E., Cotillo I., Fiandor J. M., Francisco A. F., Grimsditch D., Keenan M., Kelly J. M., Kessler A., Luise C., Lyon J. J., MacLean L., Marco M., Martin J. J., Martinez Martinez M.
S., Paterson C., Read K. D., Santos-Villarejo A., Zuccotto F., Wyllie S., Miles T. J., De Rycker M. (2023). Short-course combination treatment
for experimental chronic Chagas disease. Sci.
Transl. Med..

[15] Aguilera E., Alvarez G., Cerecetto H., González M. (2019). Polypharmacology
in the Treatment of Chagas Disease. Curr. Med.
Chem..

[16] Kratz J. M., Gonçalves K. R., Romera L. M., Moraes C. B., Bittencourt-Cunha P., Schenkman S., Chatelain E., Sosa-Estani S. (2022). The translational
challenge in Chagas disease drug development. Mem. Inst. Oswaldo Cruz.

[17] Soeiro M. d. N. C., Sales-Junior P. A., Pereira V. R. A., Vannier-Santos M. A., Murta S. M. F., Sousa A. S., Sangenis L. H. C., Moreno A. M. H., Boechat N., Branco F. S. C., Holetz F. B., Ávila A. R., Pereira M. C. S. (2024). Drug screening
and development cascade for Chagas disease:
an update of in vitro and in vivo experimental models. Mem. Inst. Oswaldo Cruz.

[18] Diniz L. F., Mazzeti A. L., Caldas I. S., Ribeiro I., Bahia M. T. (2018). Outcome
of E1224-Benznidazole Combination Treatment for Infection with a Multidrug-Resistant
Trypanosoma cruzi Strain in Mice. Antimicrob.
Agents Chemother..

[19] Planer J. D., Hulverson M. A., Arif J. A., Ranade R. M., Don R., Buckner F. S. (2014). Synergy testing of FDA-approved drugs identifies potent
drug combinations against Trypanosoma cruzi. PLoS Neglected Trop. Dis..

[20] Stein C., Migliavaca C. B., Colpani V., Rosa P. R. d., Sganzerla D., Giordani N. E., Miguel S. R. P. d. S., Cruz L. N., Polanczyk C. A., Ribeiro A. L. P., Falavigna M. (2018). Amiodarone
for arrhythmia in patients
with Chagas disease: A systematic review and individual patient data
meta-analysis. PLoS Neglected Trop. Dis..

[21] Benaim G., Paniz Mondolfi A. E. (2012). The emerging
role of amiodarone and dronedarone in
Chagas disease. Nat. Rev. Cardiol..

[22] Benaim G., Sanders J. M., Garcia-Marchán Y., Colina C., Lira R., Caldera A. R., Payares G., Sanoja C., Burgos J. M., Leon-Rossell A., Concepcion J. L., Schijman A. G., Levin M., Oldfield E., Urbina J. A. (2006). Amiodarone
has intrinsic anti-Trypanosoma cruzi activity and acts synergistically
with posaconazole. J. Med. Chem..

[23] Diniz L. d. F., Caldas I. S., Guedes P. M., Crepalde G., de Lana M., Carneiro C. M., Talvani A., Urbina J. A., Bahia M. T. (2010). Effects
of ravuconazole treatment on parasite load and immune response in
dogs experimentally infected with Trypanosoma cruzi. Antimicrob. Agents Chemother..

[24] Xiong J., Lu H., Jiang Y. (2025). Mechanisms of Azole Potentiation: Insights from Drug
Repurposing Approaches. ACS Infect. Dis..

[25] Gamarra S., Rocha E. M., Zhang Y. Q., Park S., Rao R., Perlin D. S. (2010). Mechanism of the
synergistic effect of amiodarone and
fluconazole in Candida albicans. Antimicrob.
Agents Chemother..

[26] Sass G., Madigan R. T., Joubert L. M., Bozzi A., Sayed N., Wu J. C., Stevens D. A. (2019). A Combination of Itraconazole and
Amiodarone Is Highly Effective against *Trypanosoma cruzi* Infection of Human Stem Cell-Derived Cardiomyocytes. Am. J. Trop. Med. Hyg..

[27] Zheng W., Sun W., Simeonov A. (2018). Drug repurposing screens and synergistic drug-combinations
for infectious diseases. Br. J. Pharmacol..

[28] Gulin J. E., Rocco D. M., García-Bournissen F. (2015). Quality of
Reporting
and Adherence to ARRIVE Guidelines in Animal Studies for Chagas Disease
Preclinical Drug Research: A Systematic Review. PLoS Neglected Trop. Dis..

[29] Barbosa J. M. C., Pedra Rezende Y., de Melo T. G. (2022). Experimental
Combination Therapy with Amiodarone and Low-Dose Benznidazole in a
Mouse Model of Trypanosoma cruzi Acute Infection. Microbiol. Spectr..

[30] Francisco A. F., Chen G., Wang W., Sykes M. L., Escudié F., Scandale I., Olmo F., Shackleford D. M., Zulfiqar B., Kratz J. M., Pham T., Saunders J., Hu M., Avery V. M., Charman S. A., Kelly J. M., Chatelain E. (2023). Preclinical
data do not support the use of amiodarone or dronedarone as antiparasitic
drugs for Chagas disease at the approved human dosing regimen. Front. Trop. Dis..

[31] McDonald M. G., Au N. T., Rettie A. E. (2015). P450-Based
Drug-Drug Interactions
of Amiodarone and its Metabolites: Diversity of Inhibitory Mechanisms. Drug Metab. Dispos..

[32] Wessler J. D., Grip L. T., Mendell J., Giugliano R. P. (2013). The P-glycoprotein
transport system and cardiovascular drugs. J.
Am. Coll. Cardiol..

[33] b Erratum. Expet Opin. Invest. Drugs 2009, 18(12), 1967. 10.1517/13543780903475056

[34] Ishii Y., Ito Y., Matsuki S., Sanpei K., Ogawa O., Takeda K., Schuck E. L., Uemura N. (2018). Clinical Drug-Drug Interaction Potential
of BFE1224, Prodrug of Antifungal Ravuconazole, Using Two Types of
Cocktails in Healthy Subjects. Clin. Transl.
Sci..

[35] Spósito P. Á., Mazzeti A. L., de Castro K. C. M. P., Mendes P. F., Urbina J. A., Bahia M. T., Mosqueira V. C. F. (2021). Higher
oral efficacy of ravuconazole
in self-nanoemulsifying systems in shorter treatment in experimental
chagas disease. Exp. Parasitol..

[36] Zhang Y. Q., Rao R. (2007). Global disruption of cell cycle progression
and nutrient response
by the antifungal agent amiodarone. J. Biol.
Chem..

[37] Silvestrini M. M. A., Alessio G. D., Frias B. E. D. (2024). New insights into *Trypanosoma cruzi* genetic diversity, and its influence on
parasite biology and clinical outcomes. Front.
Immunol..

[38] Ferreira B. L., Ferreira E. ´R., de Brito M. V., Salu B. R., Oliva M. L. V., Mortara R. A., Orikaza C. M. (2018). BALB/c and C57BL/6 Mice Cytokine
Responses to *Trypanosoma cruzi* Infection Are Independent
of Parasite Strain Infectivity. Front. Microbiol..

[39] Roggero E., Perez A., Tamae-Kakazu M., Piazzon I., Nepomnaschy I., Wietzerbin J., Serra E., Revelli S., Bottasso O. (2002). Differential
susceptibility to acute Trypanosoma cruzi infection in BALB/c and
C57BL/6 mice is not associated with a distinct parasite load but cytokine
abnormalities. Clin. Exp. Immunol..

[40] Andrade L. O., Machado C. R., Chiari E., Pena S. D., Macedo A. M. (2002). Trypanosoma
cruzi: role of host genetic background in the differential tissue
distribution of parasite clonal populations. Exp. Parasitol..

[41] Vespa G. N., Cunha F. Q., Silva J. S. (1994). Nitric oxide is
involved in control
of Trypanosoma cruzi-induced parasitemia and directly kills the parasite
in vitro. Infect. Immun..

[42] de
Alba-Alvarado M. C., Cabrera-Bravo M., Zenteno E., Salazar-Schetino P. M., Bucio-Torres M. I. (2024). The Functions of Cytokines in the Cardiac Immunopathogenesis
of Chagas Disease. Pathogens.

[43] Machado F. S., Dutra W. O., Esper L., Gollob K. J., Teixeira M. M., Factor S. M., Weiss L. M., Nagajyothi F., Tanowitz H. B., Garg N. J. (2012). Current understanding of immunity
to Trypanosoma cruzi infection and pathogenesis of Chagas disease. Semin. Immunopathol..

[44] Reis
Machado J., Silva M. V., Borges D. C., da Silva C. A., Ramirez L. E., dos Reis M. A., Castellano L. R., Rodrigues V., Rodrigues D. B. (2014). Immunopathological aspects of experimental
Trypanosoma cruzi reinfections. BioMed Res.
Int..

[45] Andrade S. G. (1990). Influence
of Trypanosoma cruzi strain on the pathogenesis of chronic myocardiopathy
in mice. Mem. Inst. Oswaldo Cruz.

[46] Rodríguez-Angulo H., Marques J., Mendoza I., Villegas M., Mijares A., Gironès N., Fresno M. (2017). Differential cytokine profiling in
Chagasic patients according to their arrhythmogenic-status. BMC Infect. Dis..

[47] Barbosa J. M. C., Pedra-Rezende Y., Mata-Santos H. A., Vilar-Pereira G., Melo T. G., Ramos I. P., Gibaldi D., Moreira O. C., Nunes D. F., Batista M. M., Lannes-Vieira J., Daliry A., Salomão K. (2024). Preclinical
evaluation of combined
therapy with amiodarone and low-dose benznidazole in a mouse model
of chronic Trypanosoma cruzi infection. Biomed.
Pharmacother..

[48] Sathler-Avelar R., Vitelli-Avelar D. M., Elói-Santos S.
M., Gontijo E. D., Teixeira-Carvalho A., Martins-Filho O. A. (2012). Blood leukocytes from benznidazole-treated
indeterminate chagas disease patients display an overall type-1-modulated
cytokine profile upon short-term in vitro stimulation with Trypanosoma
cruzi antigens. BMC Infect. Dis..

[49] Jia J., Zhu F., Ma X., Cao Z., Li Y. X., Chen Y. Z., Li Y. X., Chen Y. Z. (2009). Mechanisms of drug
combinations:
interaction and network perspectives. Nat. Rev.
Drug Discov..

